# Supplementary material for: Coherent terahertz radiation with 2.8-octave tunability through chip-scale photomixed microresonator optical parametric oscillation
Source: Nat Commun. 2022 Aug 31;13:5123. doi: 10.1038/s41467-022-32739-6 (PMC9433451; doi:10.1038/s41467-022-32739-6)
Supplement: Supplementary file 1 — Supplementary File [file 41467_2022_32739_MOESM1_ESM.pdf]

Supplementary Information

Coherent terahertz radiation with 2.8-octave tunability through chip-scale photomixed microresonator optical parametric oscillation

Wenting Wang[1,†,*], Ping-Keng Lu[2†], Abhinav Kumar Vinod[1], Deniz Turan[2], James McMillan[1], Hao Liu[1], Mingbin Yu[4,5], Dim-Lee Kwong[4], Mona Jarrahi[2,*], and Chee Wei Wong[1,*]

[1] Fang Lu Mesoscopic Optics and Quantum Electronics Laboratory, University of California, Los Angeles, CA 90095, United States of America

[2] Terahertz Electronics Laboratory, University of California, Los Angeles, CA 90095, United States of America

[3] State Key Laboratory of Functional Materials for Informatics, Shanghai Institute of Microsystem and Information Technology, Shanghai, China

[4] Institute of Microelectronics, A*STAR, Singapore 117865, Singapore

[†] These authors contributed equally to this work.

[*] Email: wentingwang@ucla.edu; mjarrahi@ucla.edu; cheewei.wong@ucla.edu

This Supplementary Information consists of the following sections:

I. Azimuthal microresonator mode number identification and characterization

II. Tunable optical parametric sideband and terahertz signal generation experimental setups

III. Terahertz signal frequency tunability, stability, and comparison with other THz sources.

I. Azimuthal microresonator mode number identification and characterization

Figure S1a shows the setup of the swept-wavelength interferometry where a tunable laser is utilized to measure the transmission of the unbalanced Mach-Zehnder interferometer (MZI), the gas cell, and the microresonator. The frequency response of the unbalanced MZI provides the trigger signal for the data acquisition system to synchronously record the output of the two photodetectors (PD_{Cal} and PD_{Trans}). The frequency of the mode spectra of the microresonator is calibrated by the frequency markers of the gas cell. Figure S1b shows the hybridized mode spectra at the mode-crossing frequency which can be used to indicate the azimuthal mode frequency change. Based on the measured microresonator mode spectra and the transmission of the gas cell, the mode free spectral range is obtained, and the microresonator GVD is fitted with the linear fitting as shown in Figure S1c. Figure S1d shows the extracted loaded quality factor based on the

calibrated cavity frequency and linewidth. The loaded quality factor at the pump mode is 1.55×10^6 and the coupling coefficient is 0.74.

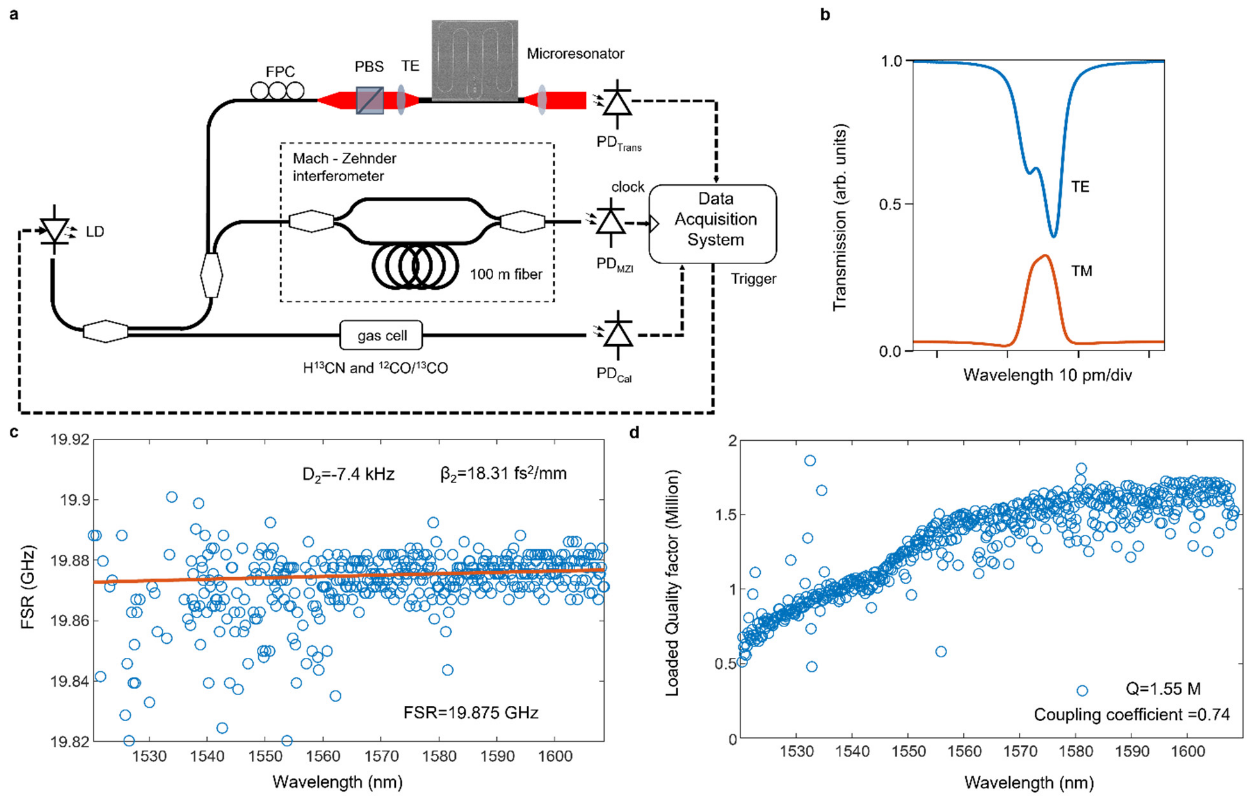

Figure S1 | Microresonator group velocity dispersion characterization setup based on swept-wavelength interferometry. a, Experimental setup for the optical transmission measurement of the microresonator to extract the wavelength-dependent quality factor and cavity mode FSR. LD: laser diode, PBS: polarization beam splitter, PD: photodetector, FPC: fiber polarization controller. **b,** Transmission of the TE and TM mode-crossing resonances. **c,** Measured microresonator GVD where $\beta_2 = 18.31$ fs^2/mm. The FSR ≈ 19.875 GHz. **d,** Measured wavelength-dependent loaded cavity quality factor where the loaded quality factor is $Q = 1.55 \times 10^6$ at the pump frequency.

In the microresonator, avoided mode-crossing is observed between TE and TM modes. The two fundamental modes experience an adiabatic crossover and the secondary resonance slowly fades away [S1]. At the crossing position, due to the superposition of the TE and TM mode, the resonance is hybridized. The frequency of the mode-crossing depends sensitively on the temperature of the microresonator [S2, S3]. The experimental setup is shown in Figure S2a where the output of the laser diode is TE-polarized and coupled into the nitride microresonator via a polarization beam splitter with a power extinction ratio of more than 30 dB. A home-made

temperature control unit (TCU) with a Peltier element is used to stabilize chip temperature to maintain the fiber-to-chip coupling. We placed the same PBS to split the TE and TM resonance spectra at the microresonator output. The light at the TE and TM polarizations is fed into two photodetectors (PDA10CF) where the outputs of the PDs are recorded by a data acquisition card simultaneously. Firstly, we increased the pump laser power via a high-power EDFA to observe the mode-pulling effect introduced by the laser heating from optical power absorption inside the nitride waveguide. Secondly, we removed the high-power EDFA and decreased the pump power to eliminate thermal effects. Then, we changed the temperature of the microresonator by controlling the TCU to observe its impact on the mode spectra. Mode-splitting is observed.

Table S1 | Calculation parameters of the avoided mode crossing.

Material parameters, symbol, unit	Parameter value
Intrinsic loss rate of mode A, γ_A	2.45×10^{-5}
Intrinsic loss rate of mode B, γ_B	3.3×10^{-6}
Coupling loss rate of mode A, Γ_A	7×10^{2}
Coupling loss rate of mode B, Γ_B	1.3×10^{-6}
Coupling strength between mode A and mode B, g_{AB} & g_{BA}	30
coupling loss rates, $\kappa_{A,B}$	1.977

The splitting frequency can be tuned due to the differential thermo-optic effects between the TE and TM modes in the birefringent microresonator. We calculate the amplitude of the splitting mode based on coupled-mode theory [S4] as shown:

$$\frac{dA}{dt} = -(i\omega_A + \gamma_A + \Gamma_A)A - ig_{BA}B - \kappa_A A_{in} \quad \text{(S1)}$$

$$\frac{dB}{dt} = -(i\omega_B + \gamma_B + \Gamma_B)B - ig_{AB}A - \kappa_B B_{in} \quad \text{(S2)}$$

where ω_A and ω_B are angular mode frequency, γ_A (γ_B) and Γ_A (Γ_B) are the intrinsic and coupling loss rates. g_{BA} and g_{AB} represent the coupling strength between the modes. The mode-splitting frequency is caused by the chip temperature change through $\omega_B - \omega_A = \alpha\Delta T$. The α is related to the difference between the TE and TM thermo-optic coefficients. The coefficients κ_A and κ_B are associated with the coupling loss rates as $\kappa_{A,B} = \sqrt{2\Gamma_{A,B}}$.

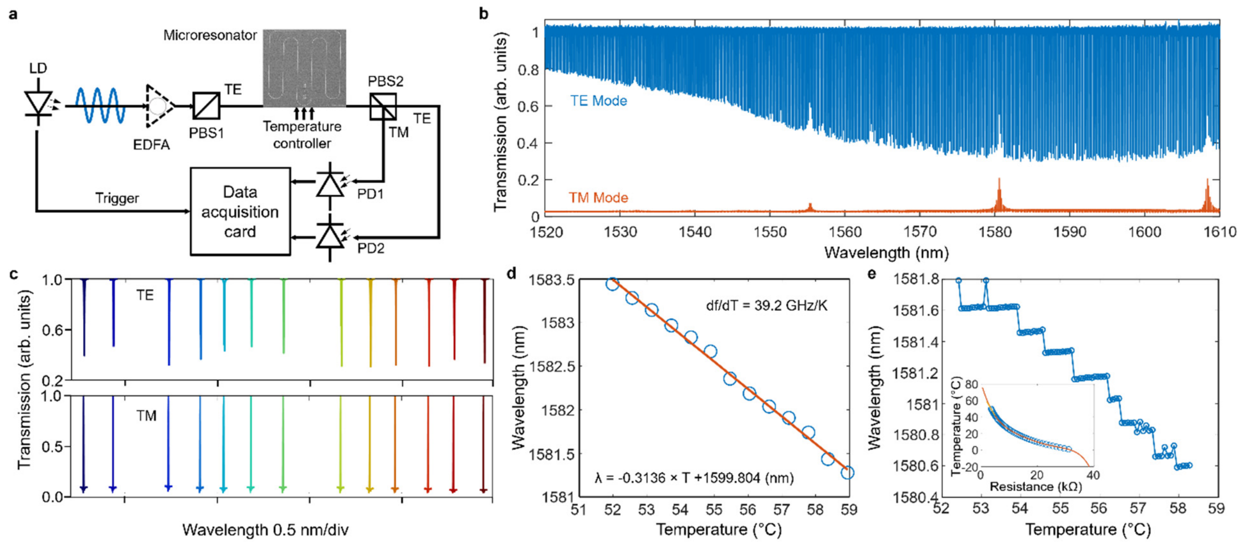

Figure S2 | Avoided mode-crossing and thermal-optic coefficient characterization. a, Experimental setup to examine mode frequency shift and avoided mode-crossing. EDFA: Erbium-doped fiber amplifier. **b,** Transmission of the microresonator at TE and TM polarizations. **c,** TM mode transmission spectra and the corresponding TE mode spectra at the different chip temperature. **d,** Measured TM resonance frequency shift depends on the chip temperature with a tuning step of 0.5 °C. **e,** Measured TM resonance frequency shift via changing the chip temperature with a tuning step of 0.05 °C.

We used the avoided mode-crossing to resolve the azimuthal order ambiguity [S5] of the measured resonances as shown in Figure S2b. The TE transmission optical spectrum shows the three dips with a wavelength separation of 26 nm which is related to the FSR difference between the TE and TM modes. The TM transmission optical spectrum shows the transmission peaks which will be an indicator for the resonant mode pulling. By changing the chip mount temperature, the resonant mode frequency can be tuned through the temperature controller with a resistance step of 0.1 kΩ. Measurements are conducted carefully to avoid temperature fluctuations. Figure S2c shows the measured TE and TM mode pulling by changing temperature. Figure S2d shows the wavelength tuning which shows the resonance wavelength and temperature dependence. Furthermore, we decreased the resistance tuning step to 0.01 kΩ to experimentally demonstrate high-resolution mode pulling as shown in Figure S2e, which shows wavelength variations originating from the temperature fluctuations. Inset is the temperature and the thermistor resistance dependence.

Table S2 | Chip-scale optical parametric oscillation (OPO).

Frequency spacing (ν_s-ν_p)	Tunability	Tuning method	Material	Nonlinear process	Device	Ref.
142 THz	No	-	Silicon nitride	$\chi^{(3)}$, dFWM	Microring	[S6]
13 THz	Yes	Different resonance	Silicon nitride	$\chi^{(3)}$, FWM-BS	Microring	[S7]
31 THz	No	-	Silicon	$\chi^{(3)}$, dFWM	Nanophotonic wires	[S8]
40.1 THz, 61.4 THz, 81.1 THz	Yes	Different resonance	Magnesium fluoride	$\chi^{(3)}$, dFWM	Microresonator	[S9]
0.64THz, 1.72THz	No	Different microring	Silicon nitride	$\chi^{(3)}$, dFWM	Microring	[S10]
330 GHz to 2.5 THz with 20 GHz frequency tuning step	Yes	Change detuning	Silicon nitride	$\chi^{(3)}$, dFWM	Microring	This work

II. Tunable optical parametric sideband and terahertz signal generation experimental setups

The frequency response bandwidth of the nanoantenna array is calibrated with two free-running tunable lasers. Figure S3a shows the terahertz frequency response calibration setup where the two lasers are combined with a 3-dB fiber coupler and amplified by an L-band optical amplifier. The amplified optical beam is focused on the nanoantenna array with a biconvex lens. The generated terahertz wave is collimated and focused with a pair of parabolic mirrors. At a chopping frequency of 20 Hz, the radiated power is measured using a pyroelectric detector with a lock-in amplifier. The measured power response is shown in Figure S3b, where multiple atmospheric absorption lines are clearly observed. Subsequently we use the nanoantenna array for frequency division of the optical parametric oscillation from mid-infrared to terahertz frequency with the detailed setup shown in Figure S3c. Figure S3d compares the detected intermediate frequency (IF) signals generated using a pair of free-running lasers (blue) and the tunable parametric oscillation (orange), showing a 3.5× frequency stability improvement for the latter case.

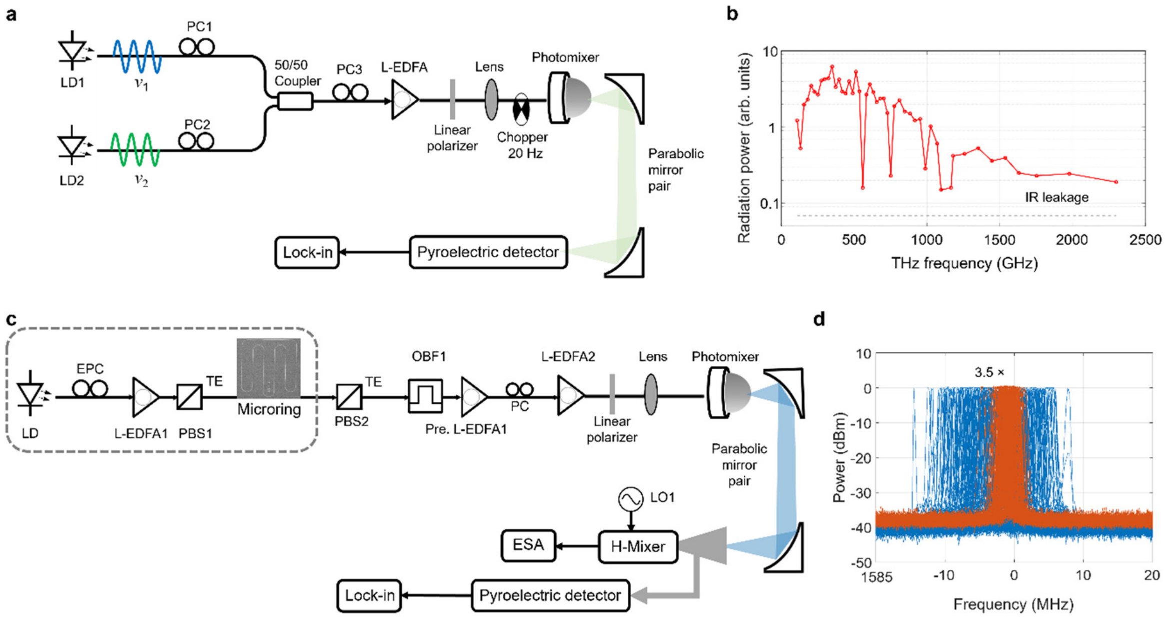

Figure S3 | Plasmonic nanoantenna array characterization with two free-running tunable lasers and the tunable optical parametric oscillation. a, Experimental setup to characterize the power response of the plasmonic nanoantenna array. PC: polarization controller, L-EDFA: L-band erbium doped fiber amplifier. **b,** Terahertz power measurement spanning from 100 GHz to 2.3 THz along with the background IR leakage detected by the pyroelectric detector. **c,** Experimental setup for detecting the terahertz radiation generated from the tunable parametric oscillation. OBP: optical bandpass filter, ESA: electrical spectrum analyzer, LO: local oscillator, H-Mixer: harmonic mixer. **d,** Detected intermediate frequency (IF) signal from a terahertz signal generated using a pair of free-running lasers (blue) and the tunable parametric oscillation (orange), showing a 3.5× frequency stability improvement for the latter case.

Figures S4a and S4b show the optical spectra of the pump and tunable parametric oscillation sidebands after power equalization when the resonant mode numbers are 9,500 and 9,501.

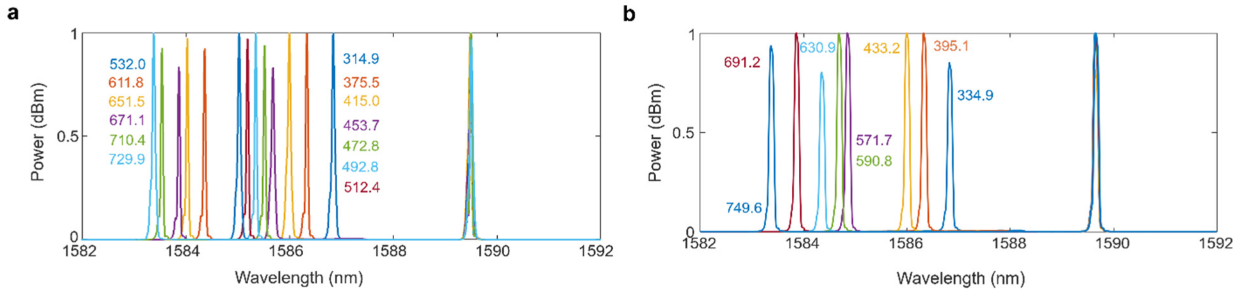

Figure S4 | Tunable optical parametric sideband. a and **b,** Measured tunable parametric

sideband when optical pumping the resonance (μ' = 9500) and the adjacent resonance (μ' = 9501). The generated frequencies (in GHz) are marked with the same color as the sidebands.

By properly optimizing the microresonator intracavity power and the pump-resonance detuning, the frequency comb cluster can be excited as shown in Figure S5a with fundamental frequency spacing of ≈ 20 GHz. The cluster optical frequency comb can be downconverted to the terahertz frequency to generate a multi-frequency terahertz oscillator. Moreover, benefiting from the local dispersion, pump-resonance detuning and the microresonator intracavity power control, the cluster frequency comb can be generated with the frequency spacing of double FSR at the different pump-signal frequency spacing as shown in Figures S5b and S5c.

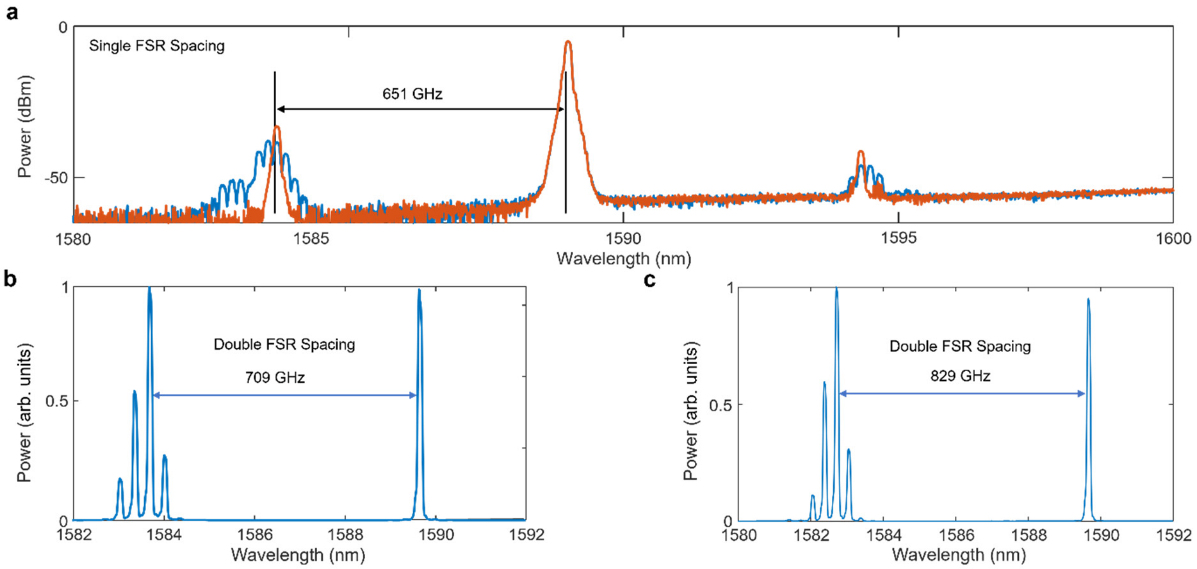

Figure S5 | Tunable optical cluster frequency combs. a, Parametric oscillation and the cluster frequency comb generated by controlling the microresonator intracavity power. **b** and **c,** Tunable optical cluster frequency combs for multi-frequency terahertz oscillators generation with pump-signal frequency spacing of 709 and 829 GHz.

III. Terahertz signal frequency tunability, stability, and comparison with other THz sources

The frequency tunability within each resonant mode is investigated by forward and backward adjusting the pump-resonance detuning. The radiation frequency can be tuned over 8.5 and 16.8 MHz as shown in Figures S6a and S6b, which is determined by phase matching condition. Then, we employ the microresonator intracavity power control to stabilize the frequency of the terahertz radiation. Figure S6c shows the frequency fluctuations before and after applying feedback locking, showing a significant improvement on the frequency fluctuations. A timing-referenced frequency

counter is used to evaluate the long-term frequency stability. Figures S6d and S6e present the measured frequency stability of the free-running and stabilized terahertz signals, which shows the root-mean-square (RMS) frequency stability of 1.28 MHz and 0.65 Hz, respectively. The corresponding frequency noise power spectral density of 10^{15} Hz^2/Hz (free-running) and 10^3 Hz^2/Hz (stabilized) at the Fourier frequency of 100 mHz are also included.

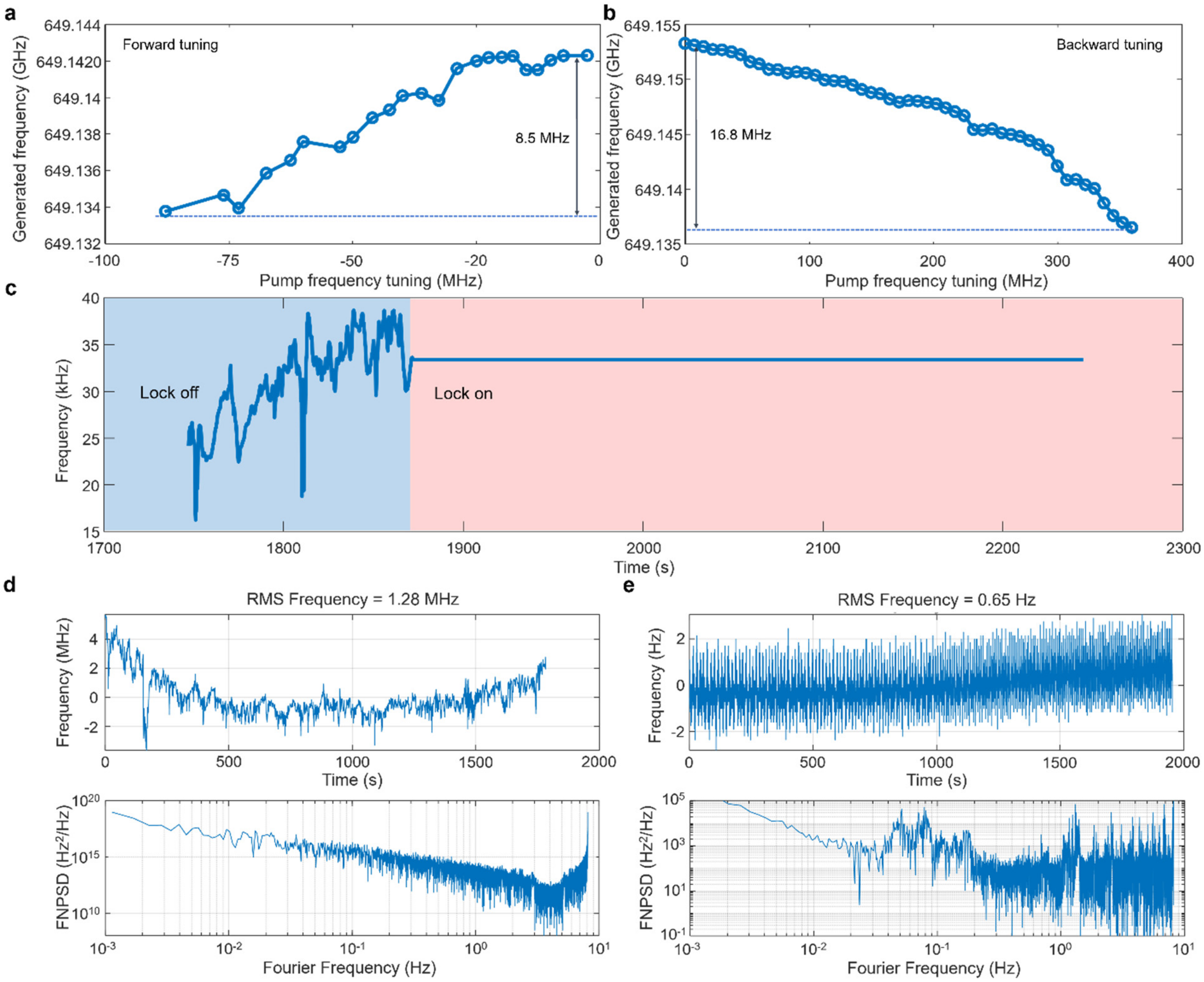

Figure S6 | Terahertz signal frequency tunability and stability. a and **b,** Center frequency tuning of the generated terahertz wave by forward- and backward-tuning of the pump laser wavelength, showing ≈ 10 MHz frequency tuning range over microresonator modes. **c,** Frequency locking by feedback control of the microresonator intracavity power showing the terahertz frequency variations before and after locking. **d** and **e,** Frequency fluctuations of the free-running and locked terahertz signal, along with the corresponding frequency noise power spectral density.

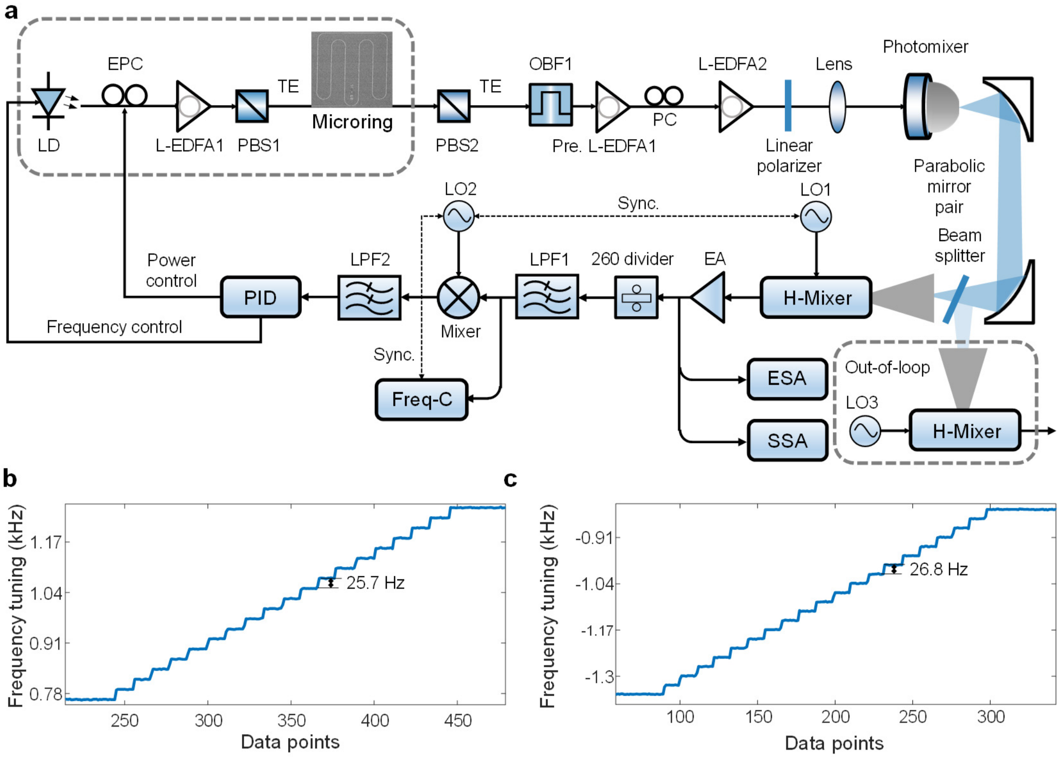

Figure S7 | Out-of-loop frequency tuning measurement. a, Frequency tuning experimental setup. **b,** In loop frequency tuning with a frequency resolution of 25.7 Hz at the carrier of 651.5 GHz. **c,** Out-of-loop frequency tuning with a frequency resolution of 26.8 Hz at the carrier of 651.5 GHz.

Figure S8 shows an example intensity fluctuation characterization with a harmonic mixer, of an optically generated 651.5 GHz signal. Due to the fast frequency fluctuations, we utilize an electrical spectral analyzer to examine the amplitude noise distribution and peak power fluctuations based on 200 measurements in the free-running condition as shown in Figure S8c. In the free-running measurement, we observe that amplitude noise reaches to the instrument-limit as shown in Figure S8b. After phase-locking the 651.5 GHz signal, we use the signal source analyzer to examine its frequency noise where we observe the characteristic noise features from the reference local oscillator and the pump laser intensity noise conversion. Since the nanoantenna array operates without any bias voltage, the direct current through the emitter is eliminated, leading to minimal heating. Consequently, the shot noise and thermal noise contribution from the nanoantenna array itself is believed to be negligible under our experimental conditions. Therefore, when the plasmonic nanoantenna array converts the incident optical frequencies into a terahertz

wave at the beat frequency, the terahertz frequency noise is dominated by the laser noise in our approach.

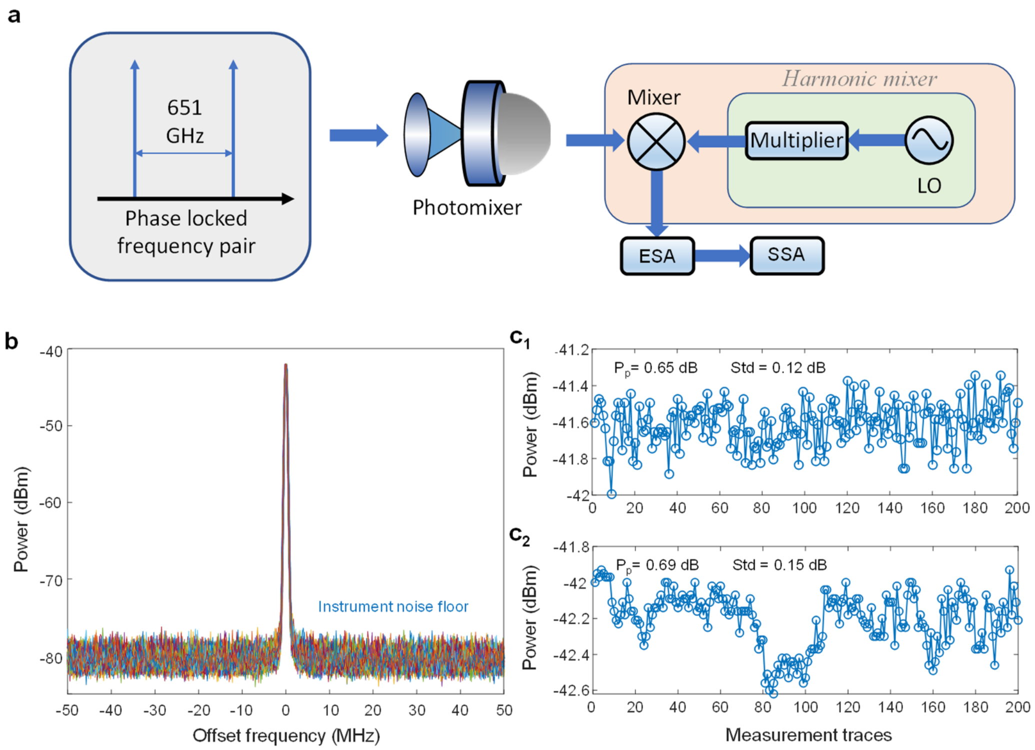

Figure S8 | Intensity fluctuation characterization with a harmonic mixer. a, Illustration of intensity and frequency noise characterization setup. **b,** RF spectral traces with continuous measurements for the phase coherent terahertz radiation after removing free-running frequency fluctuations. **c_1** and **c_2,** RF signal peak power continuous sampling for a laser pair and a phase-locked parametric oscillation.

In Table S3, we summarize the different terahertz radiation sources using integrated fully electronic, and hybrid electronic-photonic systems based on different semiconductor substrates for comparison.

Table S3 | State-of-the-art terahertz radiation sources across electronics, photonics and hybrid approaches.

Maximum frequency (f_{max}) and frequency tuning range (f_t)	Tunability (tuning step)	Linewidth	Radiation power (CW)	Material	Size	Method	Ref.
f_{max} = 1.5 THz<br>f_t = 0.61 THz	Yes	-	-	InP	8 um	25-nm InP HEMT	[S11]
f_{max} < 1.1 THz<br>f_t < 0.52 THz	Yes	-	>50mW/μm^2	InP	<6 um	InP DHBT	[S12]
f_{max} = 2.7 THz	Yes	> 100 MHz	18 μW	-	-	Schottky Diode	[S13]

f_t = 0.2 THz							
f_{max} =1.33 THz f_t = 0.15 THz	Yes	-	5.3 µW	-	0.39 mm^2	MOS-VAR	[S14]
f_{max} =0.55 THz f_t = 0.01 THz	Yes	Phase noise: -79 dBc/Hz	> 61uW	Silicon	2.16 mm^2	TPCVCO	[S15]
f_{max} =0.12 THz f_t = 0.11 THz	Yes	150 kHz	100 µW	Silicon-InP	-	Dual Tunable Lasers	[S16]
f_{max} = 2.25 THz f_t = 1.92 THz	Yes	12 kHz	10 nW	InP-Polymer	-	Dual Tunable DBR laser	[S17]
f_{max} = 0.5 THz f_t = 0.2 THz	Yes	-	3.2 µW	-	-	Spectral Shaping Frequency Comb	[S18]
f_{max} = 0.32 THz f_t = 0.22 THz	No	-	3.3 mW	SiGe	2.1 mm^2	Coupled Harmonic Oscillators	[S19]
300 GHz	No	-	-	-	-	UTC-PD	[S20]
99.75 GHz	No	-	5 mW	-	-	High-speed PD	[S21]
1.14 THz, 1.28 THz, 1.43 THz, 1.57 THz	Yes	9 kHz	600 µW	ErAs:In GaAs	-	DC-biased Plasmonic Photomixer	[S10]
f_{max} = 2.5 THz f_t = 2.2 THz	Yes (20 GHz, 27 Hz)	100 Hz	10 µW	InAs	-	Plasmonic Photomixer	**This work**

HEMT: high electron mobility transistor; DHBT: double heterojunction bipolar transistor; MOSFET: Metal oxide semiconductor field-effect transistor; UTC-PD: uni-travelling-carrier photodiode; MOS-VAR: Metal oxide semiconductor varactors; TPCVCO: triple-pushed Colpitts voltage controlled oscillator.

Supplementary References

S1. Y. Liu, Y. Xuan, X. X. Xue, P. H. Wang, S. Chen, A. J. Metcalf, J. Wang, D. E. Leaird, M. H. Qi, and A. M. Weiner, Investigation of mode coupling in normal-dispersion silicon nitride microresonators for Kerr frequency comb generation. *Optica* **1**, 137-144 (2014).

S2. X.X. Xue, Y. Xuan, C. Wang, P.-H. Wang, Y. Liu, B. Niu, D.E. Leaird, M.H. Qi, and A.M. Weiner, Thermal tuning of Kerr frequency combs in silicon nitride microring resonators. *Opt. Express* **24**, 687-698 (2016).

S3. K. Ikeda, R. E. Saperstein, N. Alic, and Y. Fainman, Thermal and Kerr nonlinear properties of plasma-deposited silicon nitride/ silicon dioxide waveguides. *Opt. Express* **16**, 12987-12994 (2008).

S4. W. L. Wang and A. N. Luiten, Mode-interactions and polarization conversion in a crystalline microresonator. *Opt. Lett.* **40**, 5431-5434 (2015).

S5. A.W. Elshaari, I. E. Zadeh, K. D. Jöns, and V. Zwiller, Thermo-optic characterization of silicon nitride resonators for cryogenic photonic circuits. *IEEE Photonics J.* **8**, 2701009 (2016).

S6. X. Lu, G. Moille, Q. Li, D. A. Westly, A. Singh, A. Rao, S.-P. Yu, T. C. Briles, S. B. Papp, and K. Srinivasan, Efficient telecom-to-visible spectral translation through ultralow power nonlinear nanophotonics. *Nature Photon*. **10**, 406–414 (2016).

S7. Q. Li, M. Davanço, and K. Srinivasan, Efficient and low-noise single-photon-level frequency conversion interfaces using silicon nanophotonics. *Nature Photon*. **10**, 406–414 (2016).

S8. X. Liu, B. Kuyken, G. Roelkens, R. Baets, R. M. Osgood Jr, and W. M. J. Green, Bridging the mid-infrared-to-telecom gap with silicon nanophotonic spectral translation. *Nature Photon*. **6**, 667–670 (2012).

S9. N. L. B. Sayson, T. Bi, V. Ng, H. Pham, L. S. Trainor, H. G. L. Schwefel, S. Coen, M. Erkintalo, and S. G. Murdoch, Octave-spanning tunable parametric oscillation in crystalline Kerr microresonators. *Nature Photon*. **13**, 701–706 (2019).

S10. S.-W. Huang, J. Yang, S.-H. Yang, M. Yu, D.-L. Kwong, T. Zelevinsky, M. Jarrahi, and C. W. Wong, Globally stable microresonator Turing pattern formation for coherent high-power THz radiation on-chip. *Phys. Rev. X* **7**, 041002 (2017).

S11. X. Mei, W. Yoshida, M. Lange, J. Lee, J. Zhou, P.-H. Liu, K. Leong, A. Zamora, J. Padilla, S. Sarkozy, R. Lai, and W. R. Deal, First demonstration of amplification at 1 THz using 25-nm InP high electron mobility transistor process. *IEEE Electron Dev. Lett.* **36**, 327–329 (2015).

S12. M. Urteaga, R. Pierson, P. Rowell, V. Jain, E. Lobisser, and M. J. W. Rodwell, 130 nm InP DHBTs with ft > 0.52 THz and fmax>1.1 THz. *In Proc. 69th Annu. Device Res. Conf.* 281–282 (2011).

S13. A. Maestrini, I. Mehdi, J. V. Siles, R. Lin, C. Lee, G. Chattopadhyay, J. Pearson, and P. Siegel, Frequency tunable electronic sources working at room temperature in the 1 to 3 THz band. *Proc. SPIE 8496*, 84960F (2012).

S14. Z. Ahmad, M. Lee and K. O. Kenneth, 1.4 THz, −13 dBm-EIRP frequency multiplier chain using symmetric- and asymmetric-CV varactors in 65 nm CMOS. *IEEE Int. Solid State Circuits Conf.* 350–351 (2016).

S15. Y. Zhao, H.-C. Lu, H.-P. Chen, Y.-T. Chang, R. Huang, H.-N. Chen, C. Jou, F.-L. Hsueh, and M.-C. F. Chang, A 0.54–0.55 THz 2×4 coherent source array with EIRP of 24.4 dBm in 65nm CMOS technology. *IEEE MTT-S Int. Microw. Symp*. 1–3 (2015).

S16. J. Hulme, M. Kennedy, R.-L. Chao, L. Liang, T. Komljenovic, J.-W. Shi, B. Szafraniec, D. Baney, and J. E. Bowers, Fully integrated microwave frequency synthesizer on heterogeneous silicon- III/V. *Opt. Express* **25**, 2422–2431 (2017).

S17. G. Carpintero, S. Hisatake, D. D. Felipe, R. Guzman, T. Nagatsuma, and N. Keil, Wireless data transmission at terahertz carrier waves generated from a hybrid InP-polymer dual tunable DBR laser photonic integrated circuit. *Sci. Rep.* **8**, 3018 (2018).

S18. X. Pang, S. Jia, O. Ozolins, X. Yu, H. Hu, L. Marcon, P. Guan, F. Da Ros, S. Popov, G. Jacobsen, M. Galili, T. Morioka, D. Zibar, and L. K. Oxenløwe, 260 Gbit/s photonic–wireless link in the THz band. *In Proc. 2016 IEEE Photon. Conf.* 1–2 (2016).

S19. R. Han, C. Jiang, A. Mostajeran, M. Emadi, H. Aghasi, H. Sherry, A. Cathelin, and E. Afshari, A SiGe terahertz heterodyne imaging transmitter with 3.3 mW radiated power and fully-integrated phase-locked loop. *IEEE J. Solid State Circuits*, **50**, 2935–2947 (2015).

S20. T. Tetsumoto, T. Nagatsuma, M. E. Fermann, G. Navickaite, M. Geiselmann, and A. Rolland, Optically referenced 300 GHz millimetre-wave oscillator. *Nature Photon*. **15**, 516–522 (2021).

S21. B. Wang, J. S Morgan, K. Sun, M. Jahanbozorgi, Z. Yang, M. Woodson, S. Estrella, A. Beling, and X. Yi, Towards high-power, high-coherence, integrated photonic mmWave platform with microcavity solitons. *Light: Sci. Appl.* **10**, (2021).
